# Genomic-based biosurveillance for avian influenza: whole genome sequencing from wild mallards sampled during autumn migration in 2022–2023 reveals a high co-infection rate on migration stopover site in Georgia

**DOI:** 10.3389/fmicb.2026.1735728

**Published:** 2026-01-28

**Authors:** Ana Papkiauri, Lela Urushadze, Tea Tevdoradze, Ketevan Sidamonidze, Giorgi Tomashvili, Mari Gavashelidze, Levan Ninua, Ivane Daraselia, Sopio Kiknavelidze, Nika Melikishvili, Bin Hu, Patrick Chain, Kaetlyn Gibson, Martha Dix, Valerie Li, Jeanne Fair, Jennifer Owen, Zurab Javakhishvili

**Affiliations:** 1 National Center for Disease Control and Public Health, Tbilisi, Georgia; 2 Center for Wildlife Disease Ecology, Ilia State University, Tbilisi, Georgia; 3 Genomics and Bioanalytics, Los Alamos National Laboratory, Los Alamos, NM, United States; 4 Modeling and Observation of Earth Systems, Los Alamos National Laboratory, Los Alamos, NM, United States; 5 Department of Fisheries and Wildlife, Michigan State University, East Lansing, MI, United States

**Keywords:** avian influenza virus, Caucasus region, coinfections, genetic diversity, migratory flyways, migratory wild birds, phylogenetic analysis, whole genome sequencing

## Abstract

The Caucasus region, including Georgia, is an important intersection for migratory waterbirds, offering potential for avian influenza virus (AIV) transmission between populations from different geographic areas. In 2022 and 2023, wild ducks were sampled during autumn migration events in Georgia to study the genetic relationships and molecular characteristics of influenza strains. Sequencing and phylogenetic analysis were used to compare the sampled strains to reference sequences from Africa, Asia, and Europe, allowing assessment of genetic relationships and virus transmission between migratory birds. Protein language modeling identified potential co-infections. Of 225 duck samples, 128 tested positive for the influenza M gene. 55 influenza-positive samples underwent whole-genome sequencing, revealing significant diversity. Analysis of the hemagglutinin (HA) segment showed notable differences among subtypes. Most samples were H6N1 and H6N6, but co-infections with combinations like H6H3, N8N1, N6H9, N2N6, and H9H6/N1N2 were also identified. These findings demonstrate the high variability of influenza viruses in migratory waterbirds in Georgia, including a notable rate of co-infections. Some samples exhibited uncommon genetic characteristics compared to other strains from the same year, suggesting Georgia’s role as a mixing vessel for influenza viruses. This facilitates reassortment during co-infections and contributes to the genetic diversity observed across flyways.

## 1 Introduction

Aquatic birds are the natural reservoir for avian influenza A viruses (AIV). Most AIVs are of low pathogenicity and cause mild or subclinical infections in aquatic birds. Low pathogenic avian influenza (LPAI) viruses are most commonly isolated from Anseriformes and Charadriiformes (Yoon et al., 2014). Since the emergence and westward spread of highly pathogenic avian influenza (HPAI) H5N1 from Southeast Asia (SE Asia), one of the outstanding questions is the role wild birds, particularly long-distance migrants, might play in the dissemination of AIV from Asia to other geographic regions. Despite considerable surveillance efforts, important knowledge gaps remain. For example, while there is increasing evidence on the contribution of migratory birds to long-distance dissemination of HPAI, our understanding of how different migratory flyways facilitate viral exchange between continents, and whether wild bird populations can sustain long-term circulation of highly pathogenic strains remains unresolved.

Active surveillance including trapping or hunting waterfowl has been a strategy applied to monitoring LPAI and HPAI (Duan et al., 2023). Active surveillance gives an opportunity for detecting avian influenza subtypes before they spread from the wild to poultry systems and then to humans, which allows for creating early intervention measures against emerging threats (AbuBakar et al., 2023; Ermonval and Morand, 2023).

To understand the epidemiology and evolution of avian influenza viruses (AIVs), a comprehensive understanding of viral composition in birds is essential. Traditionally, avian influenza virus (AIV) surveillance in wild birds relied on virus isolation in embryonated chicken eggs prior to sequencing and phylogenetic analysis (Machalaba et al., 2015; Hoye et al., 2010). However, egg-based culture is time-consuming, raises biosafety concerns, and can introduce culture-associated genetic changes, including strain competition and stochastic fluctuations in viral load, which may distort the viral population relative to the original swab sample and introduce bias into downstream analyses (Dovas et al., 2010; Verhagen et al., 2021; Hopken et al., 2021). In contrast, advances in next-generation sequencing (NGS) technologies over the past decade, including Illumina MiSeq, HiSeq, MiniSeq, and nanopore platforms, have enabled direct sequencing from clinical swabs and are now widely applied in routine AIV genomic surveillance.

This methodological shift significantly enhances our understanding of evolutionary and epizootic processes in wild bird populations. Notably, direct clinical swab sequencing improves the characterization of co-infections and the study of viral reassortment.

While phylogenetic analysis remains the gold standard for understanding evolutionary relationships between viral strains, recent advances in artificial intelligence have introduced complementary computational approaches that can enhance our understanding of viral protein function and viral evolution. Protein language models (PLMs) represent a revolutionary approach that treats amino acid sequences as a form of “language” with inherent grammatical and semantic structure (Bepler and Berger, 2021) These models, inspired by natural language processing techniques, are trained on vast databases of protein sequences to learn the underlying patterns and relationships between amino acids without relying on explicit structural information or evolutionary assumptions.

Unlike traditional phylogenetic methods that primarily focus on nucleotide or amino acid similarities to infer evolutionary relationships, PLMs can capture more complex functional relationships by identifying conserved motifs, functional domains, and interaction sites across distantly related sequences. When applied to viral proteins such as hemagglutinin (HA) and neuraminidase (NA), these models can potentially identify functional similarities that may not be apparent from sequence similarity alone, providing insights into viral adaptation and host specificity determinants.

Clustering analyses complement phylogenetic inference and protein language models by providing a dimensionality-reduction framework that facilitates intuitive visualization of complex genetic relationships. Methods such as t-SNE (t-distributed Stochastic Neighbor Embedding), a method that groups similar objects together, (Van der Maaten and Hinton, 2008) UMAP (Uniform Manifold Approximation and Projection), and hierarchical clustering can help identify natural groupings within viral populations based on genetic similarity, revealing patterns of reassortment and co-evolution across genome segments (Maaten and Hinton, 2008; McInnes et al., 2017). When applied to influenza genomics, clustering approaches can effectively identify reassortment events by revealing incongruent clustering patterns across different genome segments (Gong et al., 2021). Because it is important to be able to identify co-infections of avian innfluenza and be able to detect reassortments, using a clustering-based analysis is valuable for biosurveillance in wild birds at this critical migratory intersection in Georgia.

Immediate full-genome characterization of circulating influenza viral strains is a high priority for detection of novel and re-assorting strains and timely preventative measures against virus spread (Duan et al., 2023) Consequently, increasing efforts are now directed toward complete AIV genome sequencing directly from clinical samples (Lam and Pybus, 2018).

The Caucasus region is crossed by hundreds of thousands of migratory water birds annually. Georgia is located at the intersection of three wild bird migratory flyways – the Central Asian, East Africa-West Asia and Mediterranean/Black Sea (Lewis et al., 2013) Additionally, the wetlands within Georgia are used as a breeding, stop-over and overwintering habitat for tens of thousands of ducks offering potential for AIV transmission among bird populations originating from different geographic areas (Lewis et al., 2013). The Javakheti Upland is a volcanic plateau in southern Georgia, characterized by numerous small- and medium-sized shallow lakes. Nutrient-rich chernozem soils and shallow waters create favorable conditions for plankton and aquatic vegetation. Tens of thousands of migratory waterbirds use Javakheti lakes as migratory stopover sites during autumn as well as during spring migration. Approximately 90% of breeding waterfowl in Georgia breed on these lakes. Most of the villages in the region are located around lakes. The backyard poultry including geese, ducks and chickens are an important protein source for the local population. The domestic waterfowl have daily access to the lakes and share this habitat with wild waterfowl. Close contact among domestic waterfowl, wild breeding and migratory ducks create circumstances for coinfections, the selection of novel strains via reassortment, and increases the risk of AIV transmission from wild water birds to domestic poultry.

In this study, we utilized genomics-based biosurveillance approach to investigate the diversity and relationships of AIVs found in Georgia and determined coinfection and reassortment rates of AIV in wild birds. Specifically, we determined the avian influenza subtypes that were circulating in wild waterfowl populations during two migration seasons (Autumn 2022 and 2023) in Georgia and identified how they relate to subtypes circulating in Eurasia and Africa. We also determined the rate of co-infection with AIV in the wild bird population and showed that reassortment events can be discerned using a novel machine-learning assisted approach.

## 2 Methods

### 2.1 Sampling site selection

Knowledge of migratory waterfowl stopover ecology, particularly at wetland sites along major flyways, is crucial for understanding avian influenza virus epidemiology.

We placed our swim-in duck trap on Lake Madatapa which is situated at 2,100 meters above sea-level in the southernmost part of Georgia of the Javakheti Plateau. Surface area of the Lake Madatapa is 8.78 km^2^ and maximum depth is 1.8 meters. Migratory birds bring AIV circulating in their respective breeding populations. The optimal amounts of energy reserves and how fast these are acquired vary between species, and most likely between individuals within a species as well, and are important predictors for different migration strategies (Alerstam and Lindström, 1990; Hedenström and Alerstam, 1997). Some obligatory migrants like Garganey (Spatula querquedula), use lake for relatively short period of time (3–4 weeks) and others like Eurasian Teal (*Anas crecca*) arrive to the lake at the end of august and stay until lake is frozen, at the end of November. Both species are long distance migrants, which breed in northern Europe and western Siberia and overwinter in the Middle east and Africa. Madatapa Lake attracts migratory waterfowl from a geographically wide breeding range and is a great natural “laboratory” for investigating the role of the wild birds as vectors for AIV. The lake is also home for the large number of breeding waterfowl. When ducklings develop full juvenile plumage, locally bred ducks are starting to gather in large pre migratory aggregations. Large proportion of naïve juveniles in the pre migratory flocks creates fruitful circumstances for the spread of AIV. During this period of pre migration, there is the aggregation of birds between the local breeding ducks and arrival of long-distance migrants. During this time ducks from local breeding and migratory populations feed together.

### 2.2 Sampling

We sampled ducks during two autumn migration events in 2022 and 2023. A swim-in duck trap (‘Swedish trap’) (Bub, 1995) was used to capture various duck species. To attract wild ducks, five domestic mallards (*Anas platyrhynchos domesticus*) were placed inside the trap. These domestic ducks also served as sentinels, as they become naturally infected through contact with wild birds. Alongside wild ducks, we sampled the domestic mallards to capture the full diversity of AIV at the site. Both domestic and wild ducks were sampled twice weekly. During each sampling event, we identified the species, sex, and age of the birds, applied rings, and collected cloacal and oropharyngeal swabs. Oropharyngeal and cloacal swab samples were collected using regular-size sterile dry rayon swabs with plastic applicators (individually packaged in peel pouches) and immediately placed into RNAlater for preservation We have collected samples in total from 225 ducks (Bub, 1995).

All animal sampling procedures conducted in this study were approved by the relevant Institutional Animal Care and Use Committee (IACUC) and the Animal Care and Use Review Office (ACURO). Field sampling and handling of wild birds were performed in accordance with institutional guidelines, national regulations, and international standards for the ethical use of animals in research.

### 2.3 PCR diagnostics

The RNA extraction process was executed using the Thermo KingFisher Sample Purification System according to the manufacturer’s instructions. Subsequently, real-time reverse transcription PCR (real-time RT-PCR) assays employing TaqMan probe-based chemistry were performed on all samples to detect the influenza A virus matrix (M) gene, following the protocol described by Nagy et al. (2021). The subsequent detection of the matrix gene (M) was performed on a QuantStudio™ Real-Time PCR machine, utilizing the QuantiNova Probe RT-PCR kit in accordance with the manufacturer’s instructions.

The World Organization for Animal Health (WOAH) identifies avian influenza subtypes H5 and H7 as the primary strains of concern owing to their high pathogenicity and substantial implications for animal health. Therefore, A total of 225 cloacal swab samples were received at the National Center for Disease Control (NCDC) for research diagnostic purposes all samples were initially screened for the influenza A matrix (M) gene using real-time RT-PCR. 128 samples that were positive for the matrix gene were subsequently tested for H5 and H7 subtypes using H5- and H7-specific real-time RT-PCR assays as described by Spackman et al. (2002). However, all samples yielded negative results for H5 and H7. Subsequently, M gene–positive samples with Ct values between 22 and 30 were selected for whole-genome sequencing (WGS) using the amplification method described above, based on the SuperScript® III One-Step RT-PCR system with Platinum® Taq High Fidelity DNA Polymerase.

### 2.4 AIV genome sequencing

A genomic analysis was conducted to identify influenza strains and genetic variations, as well as to determine their molecular characteristics. Furthermore, up to 55 influenza-positive samples from wild birds were selected for further amplicon-based whole-genome sequencing based on Ct values. The AIV whole genome amplification was carried out according to (Zhou et al., 2009).

To study the genetic relationships and molecular characteristics of influenza strains circulating in wild birds, a phylogenetic analysis was performed. This involved comparing our sequences with reference sequences from various influenza strains, circulating in migratory birds from Africa, Asia, and Europe. For genetic analysis, we utilized CLCbio software tools (CLC Bio LLC, Cambridge, Massachusetts, United States) to create consensus sequences for individual samples and genes. Phylogenetic analyses of the hemagglutinin (HA) and neuraminidase (NA) genes were conducted using Geneious software (Geneious, 2025).

Sequencing of successfully amplified samples was conducted using the Illumina MiSeq sequencing platform, utilizing 301 × 2 cycles. Prior to sequencing, DNA libraries were prepared using the Illumina DNA Library Prep kit (Cat# 20060059). For genetic analysis, we employed CLCbio software tools to produce consensus sequences for individual genes. Samples were subjected to quality control, with sequences shorter than 100 base pairs being trimmed. After the trimming step, sequences were mapped to the reference of avian influenza viruses for all segments. For HA and NA identification, reference data from NCBI were retrieved, and our data were mapped onto it. Reference sequences used for HA and NA mapping are listed in Supplementary Table 3 (GenBank accession numbers). Consensus sequences were then generated from this mapping, which were subsequently subjected to BLAST analysis on NCBI for further characterization.

Reference FASTA files for H6N1 and H6N6 influenza subtypes were downloaded from diverse regions in Asia, Europe, and Africa spanning 2015 to 2024 via GISAID (Elbe and Buckland-Merrett, 2017), (Shu and McCauley, 2017). Phylogenetic analyses of the HA and NA genes were conducted using Geneious prime software Ver 2023.0, (Geneious, 2025). sequence alignment was performed using MUSCLE software to compare our samples with reference sequences (Edgar, 2004). Phylogenetic trees were constructed using maximum likelihood with 500 bootstrap replicates to assess evolutionary relationships and genetic divergence among the studied influenza subtype.

### 2.5 Data processing

Two data sources Georgian sequences derived from samples collected and sequenced as part of this study and GenBank were used in the clustering analysis. The 29 HA and 18 NA Georgian DNA sequences were converted to protein sequences. Each sequence had the subtypes for co-infections specified. The GenBank data consisted of 215,025 HA and 162,045 NA protein sequences prior to cleaning of the data (Sayers et al., 2024). After removal of missing data, duplicate entries, and subtypes that were unclear, 81,547 HA and 53,630 NA sequences remained. Further cleaning simplified hosts to be more generic (e.g., chicken, goose, etc. simplified to bird) and locations to the continental level. To combine the Georgian and GenBank datasets, this further cleaning was also applied to the Georgian data. Final columns present in the combined dataset include the sequence identifier, HA or NA subtypes, host, location, collection date, sequence, and dataset (GenBank or Georgia). Distance-based analyses of Georgian AIV sequences were conducted using MEGA software, calculating pairwise nucleotide p-distances with pairwise deletion to quantify genetic divergence within and between clades (Tamura et al., 2021).

### 2.6 Generating ESM2 embeddings

The evolutionary Scale Model ESM2, (Lin et al., 2023) was used to generate numerical embeddings for the analysis of hemagglutinin (HA) and neuraminidase (NA) sequences using language models. The CLS (classification) embedding from the last layer of the ESM2 model captures a summary of the entire protein sequence’s meaning and can be used as a condensed version of the sequence’s key information. It is designed to represent the whole sequence in a single vector, making it useful for tasks like predicting structure or function. We utilized the 8 M parameter ESM2 model from HuggingFace, esm2_t6_8M_UR50D, with no additional training on avian influenza data. The combined datasets containing either HA or NA sequences were processed in this way and saved into parquet files for efficient storage, along with the sorted meta data including sequence identifier, HA or NA serotype reported by PCR, host, location to make datasets for clustering analysis.

Samples with 50 or fewer sequences or without identified HA and NA serotypes were filtered out of the GenBank dataset. To prevent over-representation of dominant hosts, 50 sequences per serotype were selected from the GenBank dataset by random sampling across hosts, with remaining slots then distributed to hosts with surplus available sequences. The final downsampled dataset consisted of the retained Georgian dataset alongside the downsampled GenBank dataset for either HA (729 total sequences) or NA (468 total sequences). In subsets for each of the downsampled datasets, we retained all Georgian sequences and isolated specific serotypes from the GenBank sequences to match those present in the Georgian dataset: H6, H9, and H11 for HA (179 total sequences), and N1 and N6 for NA (118 total sequences).

### 2.7 Embedding clustering

Our goal was to determine whether protein language models could accurately group influenza sequences by their subtype without relying on traditional sequence alignment. To achieve this, we needed to: (1) reduce the high-dimensional embeddings to a visualizable format, (2) identify natural groupings in the data, and (3) validate that these groupings matched known serotypes.

The embeddings from the downsampled NA and HA datasets were first processed with t-Distributed Stochastic Neighbor Embedding for dimensionality reduction (van der Maaten and Hinton, 2008). t-SNE converts the complex, multi-dimensional protein embeddings into two-dimensional maps where similar sequences cluster together visually—think of it as creating a “map” where related sequences are neighbors. The perplexity parameter, which controls how the algorithm balances local versus global structure in the data, was manually tuned for each dataset to achieve clear visual separation between subtypes.

Next, we needed an objective method to define cluster boundaries rather than relying on visual interpretation. We applied the Hierarchical Density-Based Spatial Clustering of Applications with Noise (HDBSCAN) algorithm (McInnes et al., 2017). HDBSCAN was selected over traditional methods like k-means because it automatically determines the number of clusters, identifies outlier sequences that do not fit established subtypes, and generates stable clusters without requiring extensive parameter optimization.

To ensure our clusters were both internally coherent and biologically meaningful, we evaluated two complementary metrics:

- Adjusted Rand Index (ARI): Measures whether our computational clusters match the known serotype labels (values range from negative to 1; 1 = perfect agreement with known subtypes, 0 = random assignment, negative = worse than random).
- Silhouette Coefficient (SC): Measures how well each sequence fits within its assigned cluster versus neighboring clusters (values range from negative to 1; 1 = clearly belongs to one cluster, 0 = ambiguous between clusters, negative = likely misassigned).

**Figure 1:**
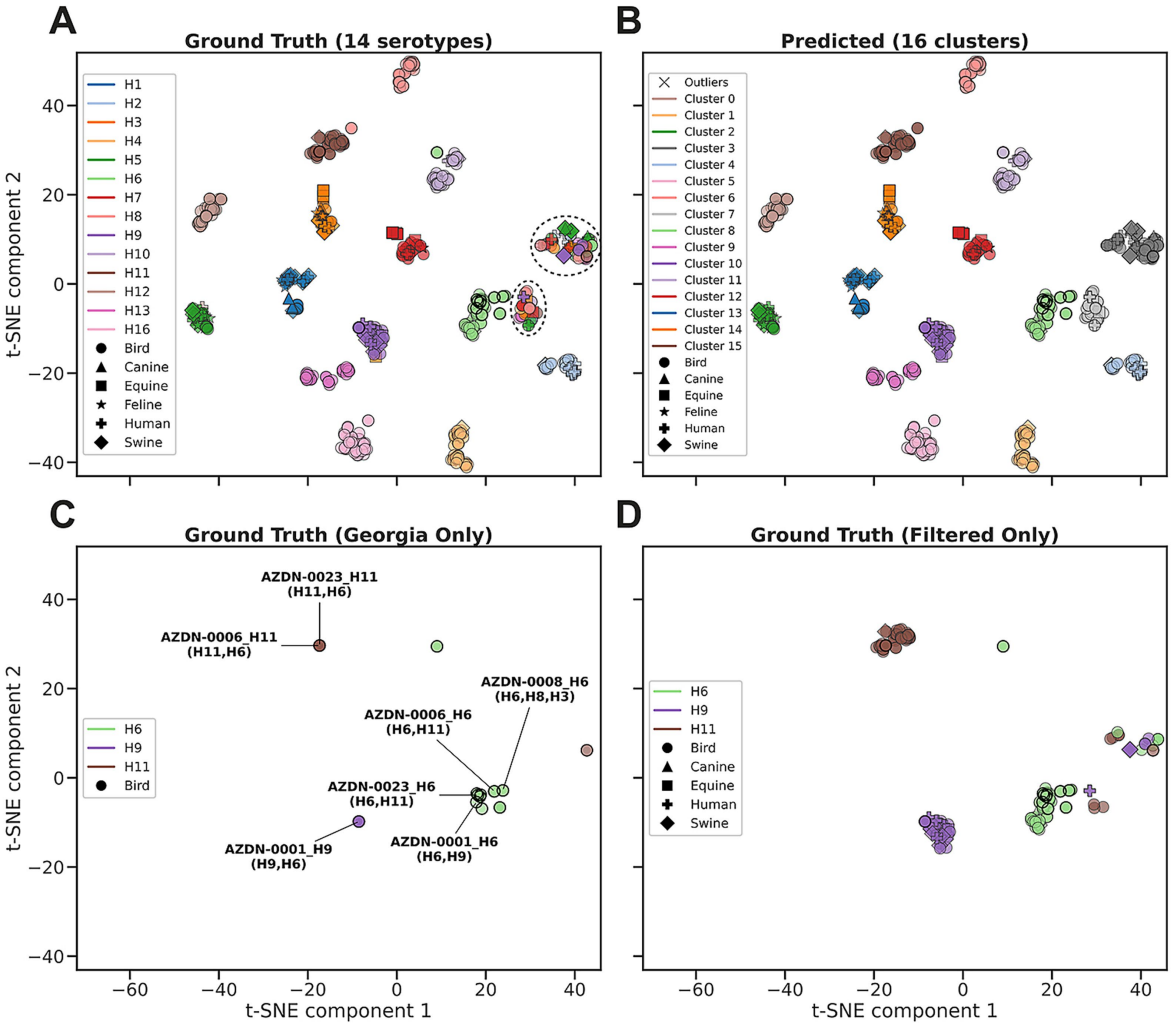
Cluster analysis of sequence-level latent representations derived from ESM2 embeddings for HA serotypes of the downsampled HA dataset. **(A)** Ground truth visualized with t-SNE, with clear separation between a majority of the serotypes. The dashed ovals indicate two heterogeneous clusters. **(B)** Predicted clusters identified by the HDBSCAN algorithm applied to the t-SNE embeddings. 16 clusters were identified with minimal outliers (black “X”), with most clusters corresponding to a dominant serotype except for clusters 3 (gray) and 7 (light gray). **(C)** Georgian ground truth data points, also highlighted with black outlines in all plots. Annotated points are Georgian samples associated with multiple serotypes (listed in parentheses), with the primary serotype stated in the identifier. **(D)** All H6 (light green), H9 (purple), and H11 (brown) sequences filtered from the ground truth. Marker shapes indicate the host species.

We systematically tested combinations of HDBSCAN parameters (min_sample_size and min_samples), which control the minimum density required to form a cluster. Parameter optimization was stopped after achieving an ARI of 0.76 and SC of 0.77 (Figure 1), indicating that our clusters both aligned well with known serotypes and were internally cohesive.

## 3 Results

Of the 225 duck samples collected, 128 tested positive for the influenza M gene. From these, 55 were selected for whole-genome sequencing. Most samples were H6N1, with some H6N6, but several cases of co-infection were also detected. Samples collected in 2022 were identified as H6N1, H6N6, H9N2, H4N6, H2N1, and H6N9. In 2023, collected samples were identified as H6N1, H11N9, H3N8, and H1N3. Furthermore, during 2022–2023, among the 55 positive samples, a total of 5 coinfections were identified. In 2022 samples with co-infections such as H9 N2 N6, and H9 H6 N1 N2 were identified. In 2023, samples with following co-infections have been identified H8 H6 H3 N1 N6, H11 H6 N9 and H11 H6 N6 N1. The avian influenza virus sequences generated in this study were deposited in the GISAID database. All high-quality genome segments included in the phylogenetic and clustering analyses were submitted, with accession numbers ranging from EPI_ISL_20144693 to EPI_ISL_20144715.

**Figure 2:**
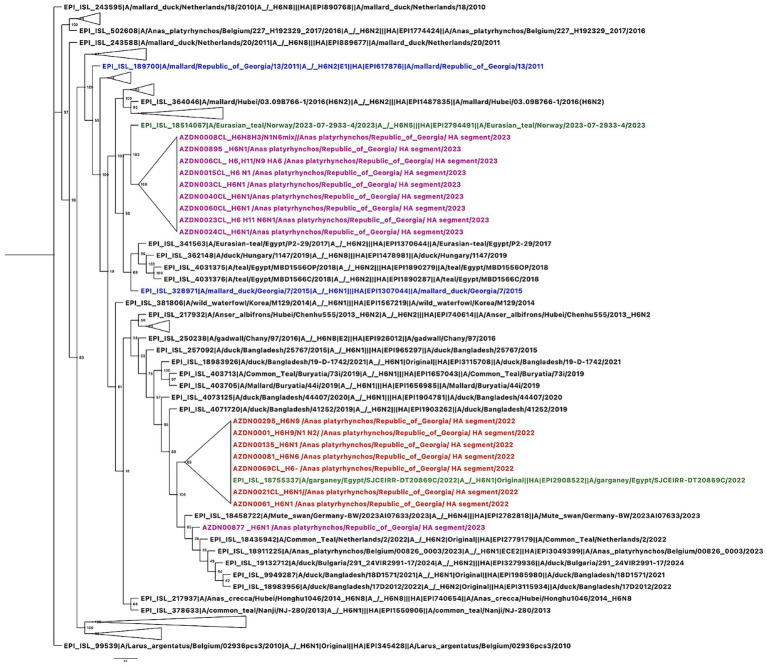
Phylogenetic tree based on the HA gene of H6 influenza viruses isolated from wild mallards during the autumn migration in Georgia, 2022–2023. Phylogenetic tree of HA gene was performed with the GTR GAMMA nucleotide model using RAxML with 500 bootstrap replicates. The isolate names in the tree are color-coded according to the year of isolation. The reference viruses obtained in this study are highlighted in black, representative strains isolated in Georgia in 2022 are highlighted in orange, samples isolated in 2023 are highlighted in purple, and Georgian isolates identified prior to 2022 are shown in blue, and the most closely related non-Georgian strain is shown in green.

**Figure 3:**
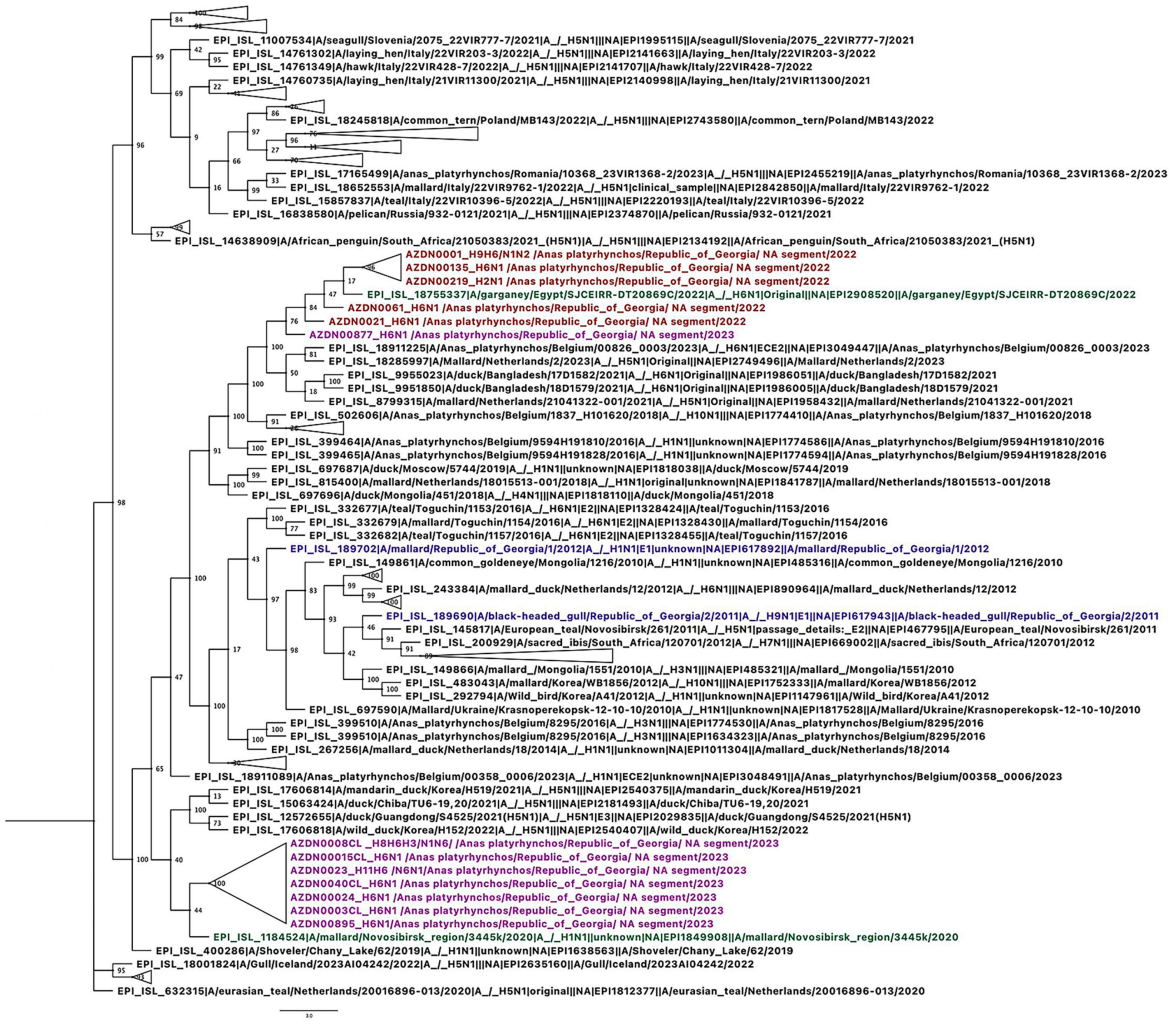
Phylogenetic tree based on NA gene of the N1 avian influenza. Phylogenetic tree of NA gene was performed with GTR GAMMA nucleotide model using RAxML with 500 bootstrap replicates. The isolate names in the tree are colored according to the year of isolation. The reference viruses obtained in this study are highlighted in black, representative strains isolated in Georgia in 2022 are highlighted in orange, and samples isolated in 2023 are highlighted in purple. Georgian isolates identified prior to 2022 are shown in blue and the most closely related non-Georgian strain is shown in green.

**Figure 4:**
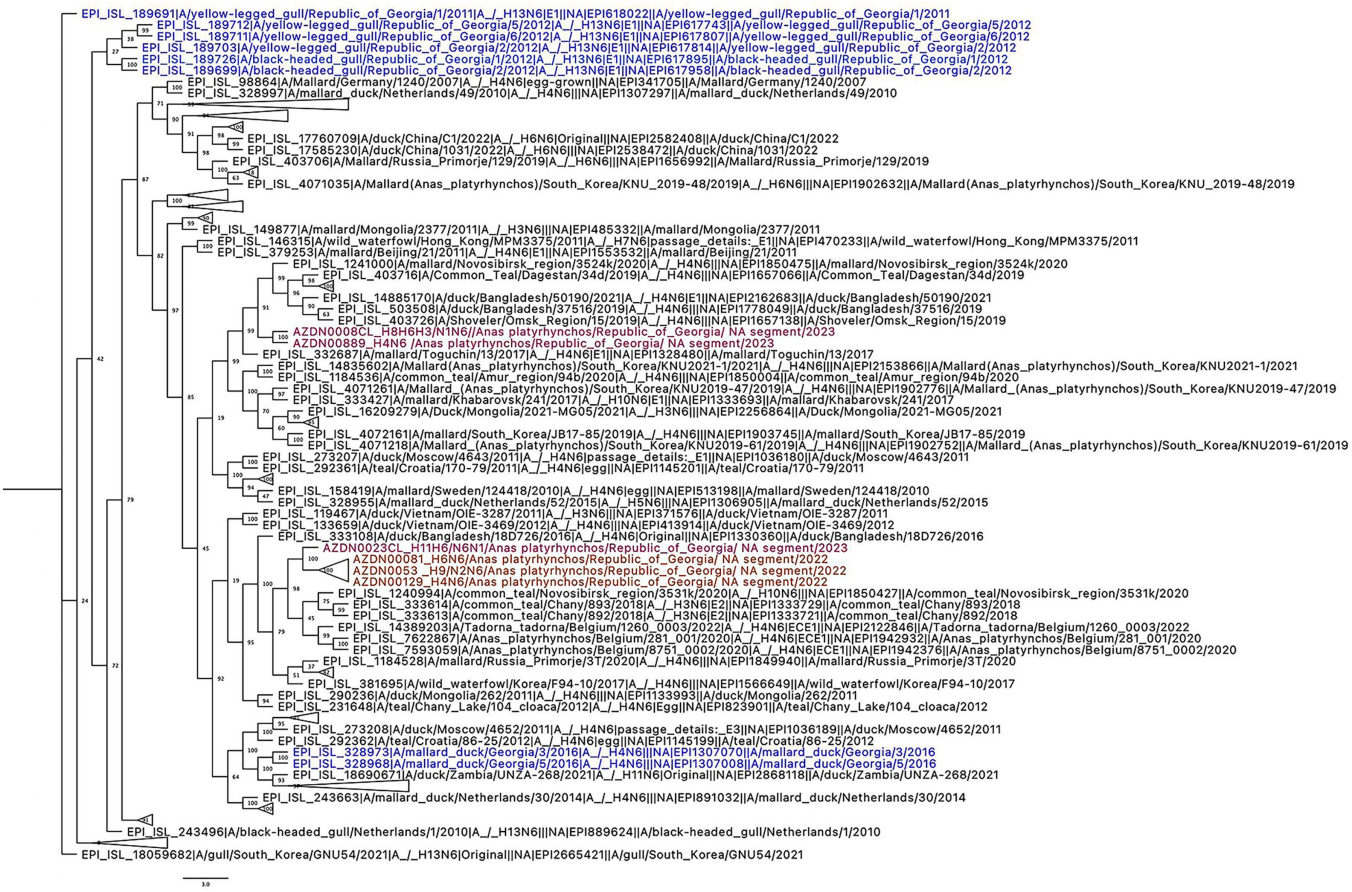
Phylogenetic tree based on NA gene of N6 avian influenza. Phylogenetic tree of NA gene was performed with GTR GAMMA nucleotide model using RAxML with 500 bootstrap replicates. The isolate names in the tree are colored based on the year of isolation: reference viruses from this study are highlighted in black, representative strains isolated in Georgia in 2022 are indicated in orange, and samples isolated in 2023 are represented in purple. Georgian isolates identified prior to 2022 are shown in blue.

Phylogenetic analysis of the HA gene revealed that in 2022, H6 viruses circulating in migratory waterbirds in Georgia belonged to a single clade closely related to viruses previously isolated in North Africa and Europe. In contrast, the H6 viruses detected in 2023 grouped into two distinct clades. One clade corresponded to the lineage observed in 2022, while the majority of the 2023 H6 isolates clustered within a separate clade, which was more closely related to viruses circulating in Europe (Figure 2). These findings suggest that diverse lineages of H6 viruses were present in Georgia in 2023 and indicate that at least two distinct H6 lineages circulated in the region, likely introduced via different migratory flyways. Similarly, phylogenetic analysis of the NA gene showed that in 2022, N1 viruses circulating in Georgia also belonged to a single clade closely related to viruses reported from North Africa and Europe. In 2023, however, N1 viruses from the study site grouped into two distinct clades. One clade corresponded to the lineage observed in 2022, while most of the 2023 N1 isolates clustered within a separate clade that was more closely related to viruses isolated from the Novosibirsk region in southern Siberia (Figure 3). In 2022, N6 viruses circulating in Georgia, belonged to one clade and were closely related to the viruses isolated in Central Siberia and Europe. In 2023, N6 viruses isolated from our study site belonged to two distinct clades. One was the same clade as in 2022, but most of the N6 viruses belonged to the other, distinct clade closely related to the viruses isolated from southern part of Siberia and southern part of Asia (Figure 4). Pairwise nucleotide distances (p-distance; pairwise deletion in MEGA) were calculated among the Georgian sequences analyzed in this study and quantified the lineage structure observed in the phylogenies. For H6-HA, within-clade divergence among Georgian samples was low (0.101–0.419%), whereas mean between-clade divergence was 4.658%, indicating the presence of two clearly separated Georgian H6-HA lineages. N1 showed a comparable pattern, with low within-clade divergence (0.013% in the predominantly 2023-like Georgian clade and 0.593% in the predominantly 2022-like Georgian clade) and high between-clade divergence (4.296%), consistent with concordant clustering with H6-HA. In contrast, N6 exhibited weaker structure among Georgian samples, with low within-clade divergence (0.097–0.176%) and lower between-clade divergence (0.675%). Notably, the 2023 sample AZDN00877 (EPI_ISL_20144708) shows markedly lower mean nucleotide dissimilarity to the 2022 HA sequences (mean 0.765%; range 0.590–0.941%) than to the remaining 2023 HA sequences (mean 4.632%; range 4.586–4.821%), consistent with its phylogenetic placement within the 2022-like H6-HA clade rather than the main 2023 cluster.

We sought to leverage advancements in machine learning and the application of protein language models (PLMs) to further characterize the HA and NA sequences. We used the ESM2 model to generate numerical representations (hereafter referred to embeddings) for each sequence then used the HDBSCAN algorithm to cluster the sequence embedding to assign the HA and NA sequences based on similarity in the embedding space. A total of 729 HA sequences were used in the analysis (29 Georgian, 700 GenBank), with a subset of 179 sequences for the specific analysis of the H6, H9 and H11 serotypes (29 Georgian, 150 GenBank). The GenBank sequences were randomly selected as reference sequences for their reported HA and NA types. We provided these sequences to the ESM model to generate sequence embeddings that were processed by t-SNE to visualize the relationships in a 2D representation. The ground truth t-SNE plot with all the sequences color coded by their reported serotype showed a clear separation between the majority of the 14 serotypes, except for two clusters that contained a combination of all serotypes with mixed colors (Figure 1A). Using the same 2D representation, HDBSCAN predicted 16 clusters that closely matched the ground truth, with minimal outliers detected, and two additional new clusters (Clusters 3 and 7), corresponding to the heterogeneous clusters found in ground truth (Figure 1B). All Georgian co-infection sequences clustered with one of the serotypes in the co-infection (Figure 1C), reflecting which HA type was dominant in each sample. For example, AZDN-0023 and AZDN-0006 (both H11 + H6 co-infections) clustered with H11 sequences, while AZDN-0008, AZDN-0006 and AZDN-0023 (both H6 + H11 or other HA-co-infections) clustered with H6 sequences. Filtering all the HA sequences, including the reference sequences, to just H6, H9, and H11 (the only serotypes reported by PCR in the Georgian sequences), we see increased diversity in H6 co-infections (Figure 1D). It is also worth noting that some of the sequences that originated from different hosts showed high similarity (Figures 1A,D), which indicate cross host infection possibilities.

**Figure 5:**
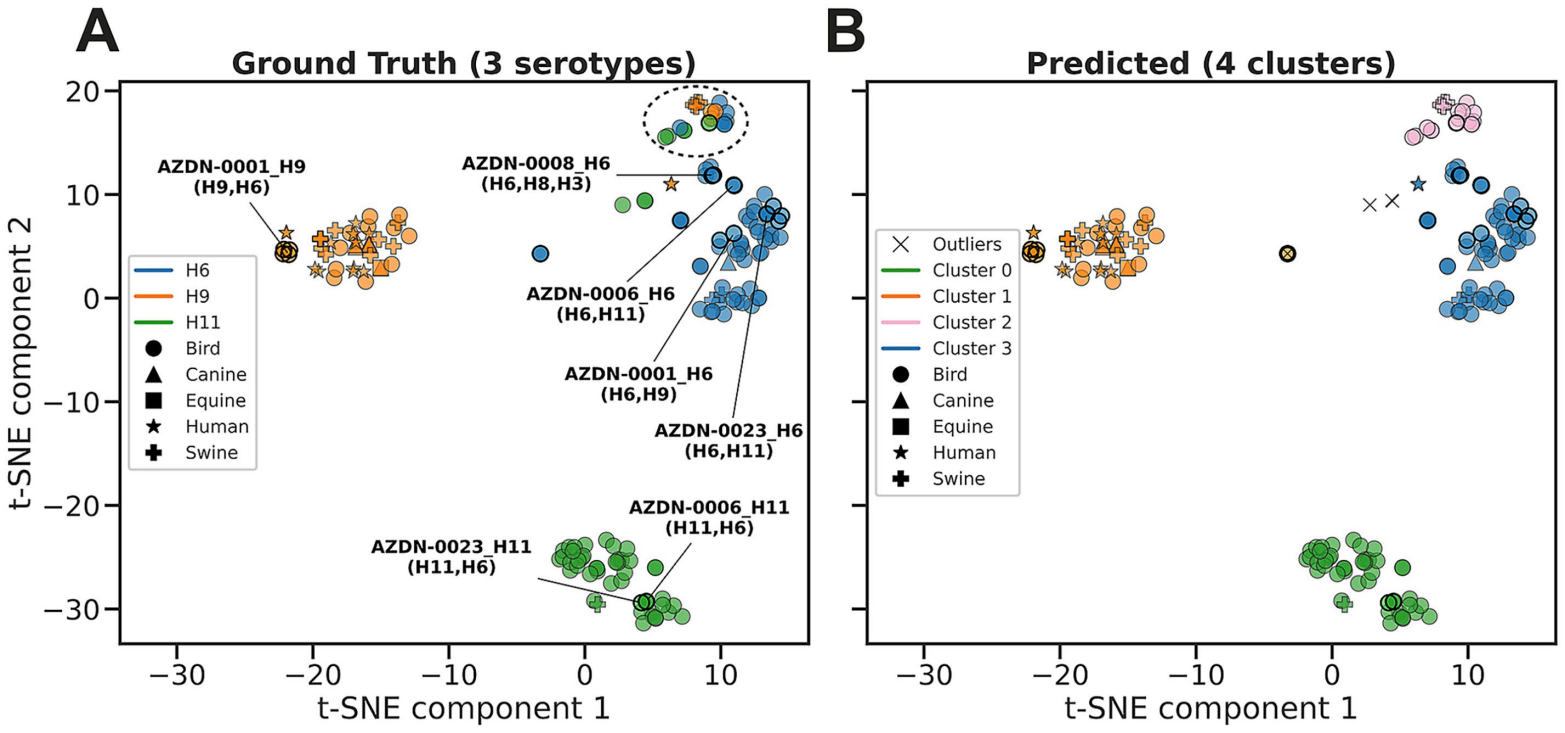
Cluster analysis of sequence-level latent representations derived from ESM2 embeddings for H6, H9, and H11 serotypes subset from the downsampled HA dataset. **(A)** Ground truth visualized with *t*-SNE, with mostly clear separation between the H6 (blue), H9 (orange), and N6 (green) clusters. Annotated points are Georgian samples associated with multiple serotypes (listed in parentheses), with the primary serotype stated in the identifier. The dashed oval indicates a heterogeneous cluster. **(B)** Predicted clusters identified by the HDBSCAN algorithm applied to the *t*-SNE embeddings. Four clusters were identified, with three that closely correspond to the *t*-SNE clusters with minimal outliers (black “X”): cluster 0 (green) with H11, cluster 1 (orange) with H9, and cluster 3 (blue) with H11. Cluster 2 (light pink) consists of multiple serotypes. Marker shapes indicate the host species. Georgian data points are highlighted with black outlines.

For a deeper analysis, t-SNE and HDBSCAN were performed on a subset of the serotypes of interest, H6, H9, and H11, to remove the background of other HA types. An ARI (measuring clustering accuracy against known serotypes, where 1.0 indicates perfect agreement) of 0.75 and a SC (measuring cluster quality where 1.0 indicates ideal cluster assignment) 0.66 were observed as the best scores, suggesting clustering close to the ground truth, with slightly less distinct clustering as seen in Figure 1. The ground truth showed mostly clear separation with minimal outliers between the 3 serotypes, with one small cluster with a mix of all three serotypes (Figure 5A). HDBSCAN predicted 4 clusters identifying the mixed serotype cluster as its own, cluster 2 (Figure 5B), which may indicate either drastic changes in the composition of the mixed infection or significant sequence changes in these genes.

**Figure 6:**
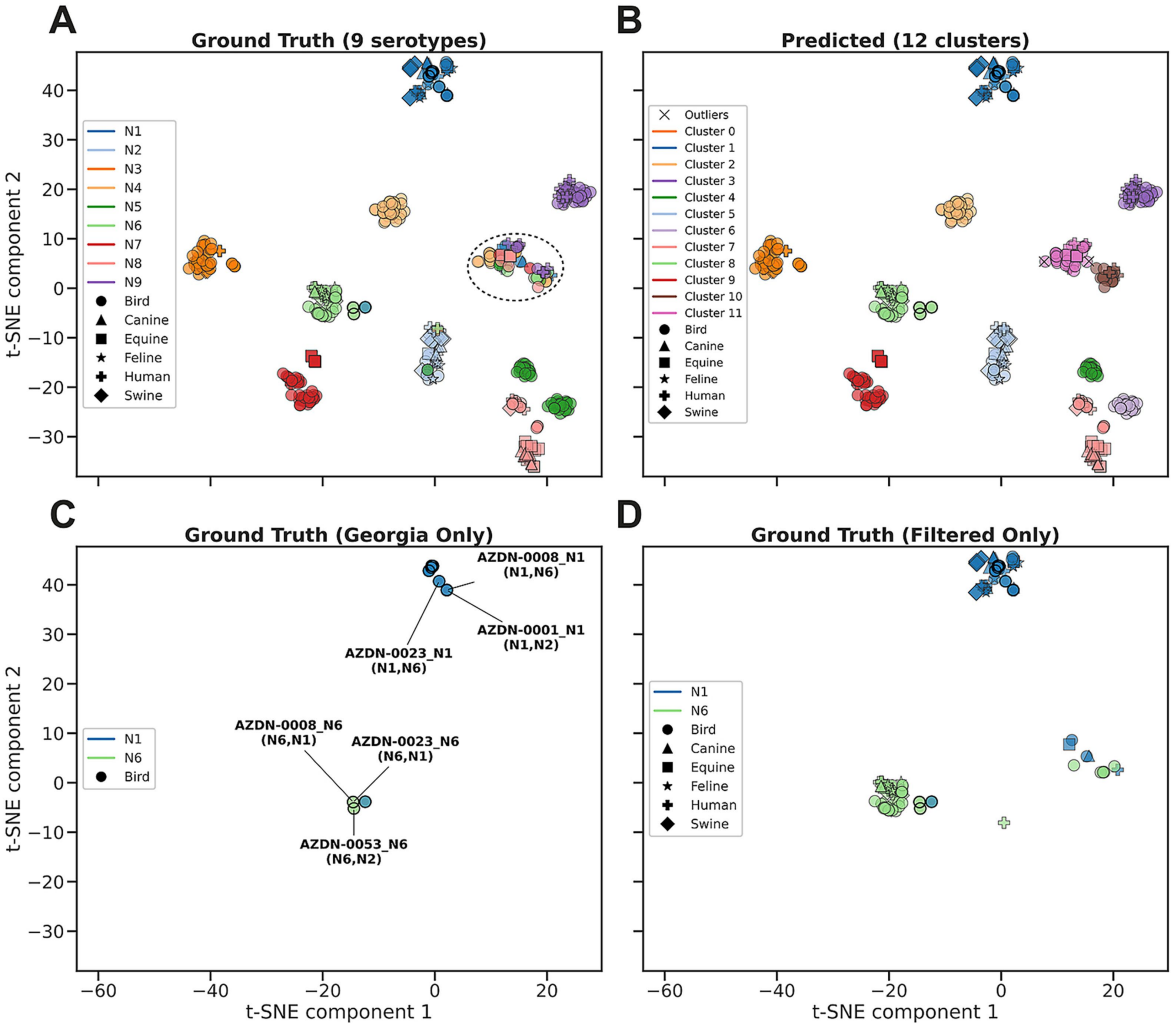
Cluster analysis of sequence-level latent representations derived from ESM2 embeddings for NA serotypes of the down sampled NA dataset. **(A)** Ground truth visualized with t-SNE, with mostly clear separation between the serotypes. The dashed oval indicates a heterogeneous cluster. **(B)** Predicted clusters identified by the HDBSCAN algorithm applied to the t-SNE embeddings. Twelve clusters were identified with minimal outliers (black “X”), with clear sub-clusters corresponding to N5 in clusters 4 (green) and 6 (light purple). **(C)** Georgian ground truth data points, also highlighted with black outlines in all plots. Two distinct clusters can be observed for the N1 (blue) and N6 (light green) serotypes, except for one N1 point located near the N6 cluster (AZDN-0021_N1). Annotated points are Georgian samples associated with multiple serotypes (listed in parentheses), with the primary serotype stated in the identifier. D All N1 (blue) and N6 (light green) sequences filtered from the ground truth. Marker shapes indicate the host species.

A similar analysis was performed on the NA sequences. A total of 468 NA sequences were used in the analysis (18 Georgian, 450 GenBank), with a subset of 118 sequences for the serotype specific analysis of N1 and N6 (18 Georgian, 100 GenBank). An ARI of 0.77 and a SC 0.73 were observed as the best scores, suggesting clustering close to the ground truth, with distinct clustering. The ground truth shows mainly clear separation between the 9 serotypes (Figure 6A), with an additional cluster consisting of a mix of all serotypes. HDBSCAN predicts a total of 12 clusters, with minimal outliers found near cluster 11 (Figure 6B). The additional clusters are a result of the mixed serotype cluster splitting in half and the N5 cluster from the ground truth being split along the two parts of the cluster. The ground truth containing only the Georgian data shows 2 distinct clusters along the serotype classification (Figure 6C). The ground truth including the GenBank data for N1 and N6 generally matched the 2 clusters from the Georgia-only data (Figure 6D). Some of the NA sequences also showed high similarity even though they were found in different hosts (Figures 6A,D).

**Figure 7:**
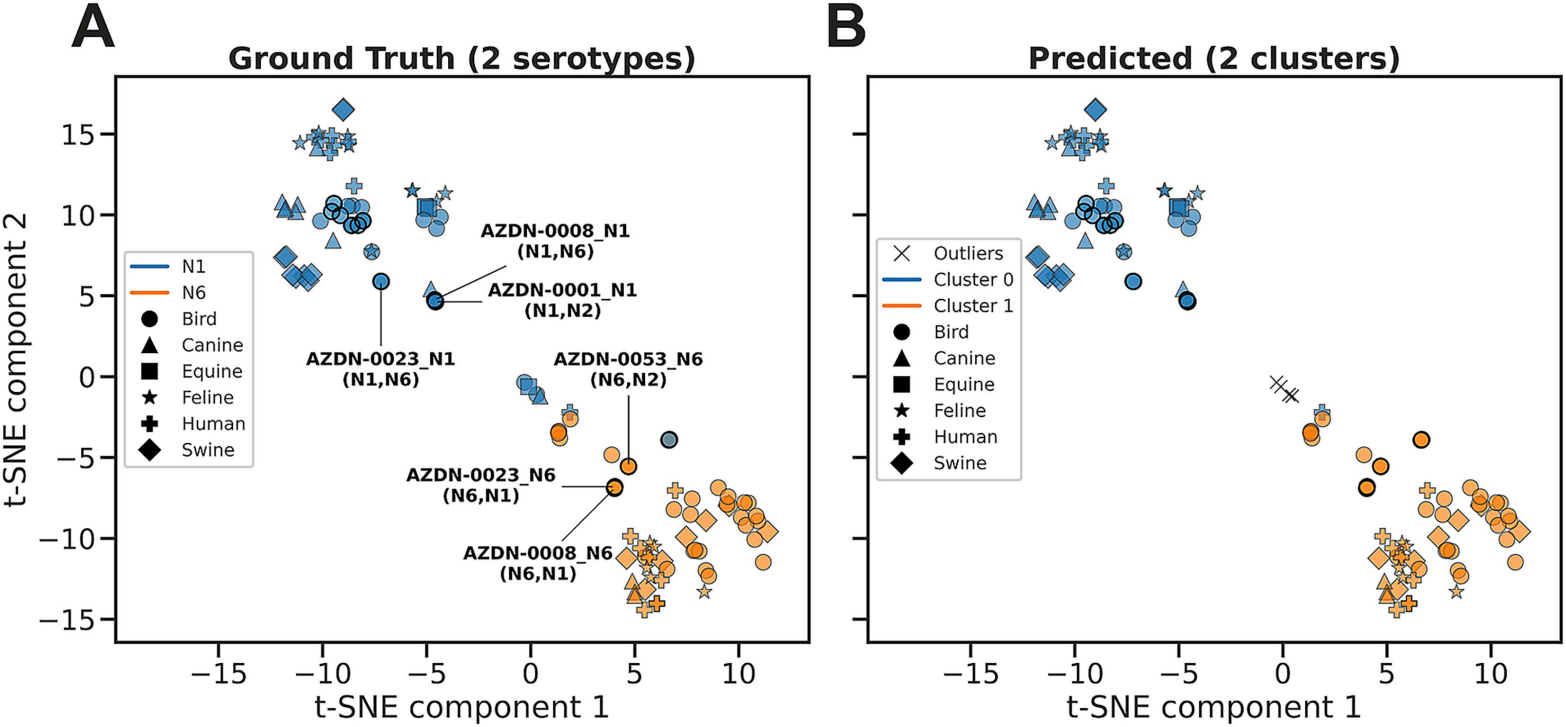
Cluster analysis of sequence-level latent representations derived from ESM2 embeddings for N1 and N6 serotypes subset from the down sampled NA dataset. **(A)** Ground truth visualized with *t*-SNE, with distinct separation between the N1 (blue) and N6 (orange) clusters. Annotated points are Georgian samples associated with multiple serotypes (listed in parentheses), with the primary serotype stated in the identifier. **(B)** Predicted clusters identified by the HDBSCAN algorithm applied to the *t*-SNE embeddings. Two clusters were identified that closely correspond to the *t*-SNE clusters with minimal outliers (black “X”): cluster 0 (blue) with N1, and cluster 1 (orange) with N6. Marker shapes indicate the host species. Georgian data points are highlighted with black outlines.

The cluster analysis was performed for just the subset of N1 and N6 sequences. An ARI of 0.90 and a SC 0.53 were observed as the best scores, suggesting near-perfect clustering to the ground truth, with decent distinction between clusters. The ground truth showed a distinct separation between the N1 and N6 data points (Figure 7A). HDBSCAN predicted 2 clusters that closely represented the clusters in the ground truth (Figure 7B). Additionally, HDBSCAN identified several outliers that were points between the clusters. There is also a point, identified as AZDN-0021_N1, that wrongly clusters with N6, suggesting a possible mislabeling of serotype (Figures 6, 7).

## 4 Discussion

To study the genetic relationships and molecular characteristics of influenza strains from wild birds and understand virus transmission between birds, a phylogenetic analysis was conducted by comparing our sequences with reference sequences from various circulating influenza strains in migratory birds from Africa, Asia, and Europe. This allowed the assessment of genetic relationships among these strains and determination of how they relate to others. Our findings in this study emphasize the extensive diversity and variability of influenza viruses, which are influenced by their geographical distribution. They underscore the viruses’ ability to evolve as they move across different regions, highlighting the importance of understanding these variations for tracking viral movement and adaptation across diverse populations. In this study, phylogenetic analyses of HA and NA genes from avian influenza viruses collected in Georgia revealed clear year-to-year differences in lineage composition. While both HA and NA genes of the 2022 isolates were associated with a single clade linked to North Africa and Europe, the viruses sampled in 2023 were more genetically diverse, forming two distinct clades. This shift highlights the dynamic nature of avian influenza virus circulation in Georgia and underscores the role of overlapping migratory flyways in shaping viral diversity across years.

Distance-based analyses of Georgian AIV sequences support the presence of two well-differentiated H6-HA lineages circulating in Georgia, with between-clade divergence (~4.7%) an order of magnitude greater than within-clade variation (≤0.419%). The same pattern observed for N1 suggests shared evolutionary histories between HA and NA segments within the Georgian dataset. In contrast, N6 showed weaker genetic structuring among Georgian samples. The placement of a 2023 Georgian isolate within the 2022-like Georgian H6-HA and N1 lineages suggests continued circulation and/or re-introduction of 2022-like viruses into the 2023 sampling period in Georgia.

The findings presented in this paper highlight the significant difference in AIV co-infection rates observed between previous study from Georgia, employing classical egg inoculation methods (Lewis et al., 2013), and this study, utilizing modern genomic methods. Our analysis indicates better sensitivity and specificity of genomic-based biosurveillance methods for the detection of AIV diversity. The higher co-infection rate observed in our study, using genomic approaches, likely reflects methodological limitations of egg inoculation. Especially it is apparent when simultaneously detecting multiple viral strains due to factors such as viral competition and replication bias within the chicken embryo. On the other hand, genomic methods play a crucial role in improving our understanding of in-host AIV diversity and evolutionary potential. The ability to accurately detect co-infections with genomic-based methods is particularly important for the monitoring of emergence of reassortant viruses. Novel reassortant viruses might exhibit altered pathogenic or transmissibility characteristics. Based on our findings, we recommend that genomic-based biosurveillance be continued and expanded for AIV surveillance programs globally. This enhanced surveillance will help to clarify a more accurate and complete picture of viral evolution and epidemiology. Improving our understanding of AIV evolution and epidemiology will ultimately enhance our preparedness for future outbreaks.

Our study demonstrates how PLM can be leveraged to monitor zoonotic pathogen sequences. The approach used here begins with protein sequence presentation, followed by dimensional reduction (t-SNE) and cluster detection, and is broadly applicable for studying closely related sequences.

The PLM approach may enable real-time biosurveillance by rapidly translating pathogen sequence data into predictions of critical phenotypic properties. For example, by connecting PLM to downstream machine learning tasks, it can help to predict binding affinity, expression levels, and cross-species transmission potential. PLMs have the potential to provide public health systems with actionable intelligence for prioritizing experimental validation efforts and identifying emerging variants that pose elevated pandemic risk before they achieve widespread circulation.

The notable increase in diversity of certain mixed infections (e.g., H6, H9, and H11) in the t-SNE plots may be explained by the fact that only the consensus sequences from Georgia were used, which were heavily influenced by the compositional ratio of different sequences. It is also possible that some of the reference sequences we used contained mixed subtypes.

There were several limitations with the PLM analysis. One, we only used a small number of reference sequences. A more detailed analysis could incorporate all the high-quality sequences from all serotypes, which would improve clustering resolution and allow more accurate analysis by different hosts. In this study, we noticed highly similar sequences found in different hosts (Figures 1, 5), which suggests these sequences may infect both human and different animals. With more reference sequences, we shall be able to develop more rigorous detection algorithms with confidence internal for sequences warranting extra attention. Second, we did not perform the fine-tuning of the PLM model using influenza sequences due to limited resources. Future plans include fine-tuning a collection of serotype specific PLM models to increase the detection accuracy of future models.

Despite these analytical limitations, the observed clustering patterns offer important insights into the biological dynamics of influenza co-infection and reassortment that may be difficult to resolve phylogenetically. Hence, it can complement traditional phylogenetic analysis based on multiple sequence alignment. It is worth exploring how to best combine these two approaches to further increase our capability for sequence-based biosurveillance.

Detection of co-infections is becoming increasingly important for understanding the evolution and spread of influenza viruses. Although AIV co-infection is predominantly reported in wild birds and poultry, serological detection of both H7 AIV and H9 AVI antibodies among farmed mink and farmed fox in Eastern China provides evidence of how these two subtypes can contribute to reassortment and cross-species transmission (Yu et al., 2020) Recurrent infection in both animals and humans leads to viral recombination, mutation, and susceptibility to co-infection (Yu et al., 2020). Infected farm workers can then introduce avian and human influenza virus strains to farm animals, like pigs, that are highly susceptible to coinfections and create excellent mixing vessels due to their mixed sialic acid receptor (AbuBakar et al., 2023).

Co-infection is a key mechanism driving reassortment, in which genetic material is exchanged between co-infecting influenza viruses, potentially leading to the emergence of novel strains with altered virulence or host range. Research indicates that co-infection can modulate the severity of disease, with some studies suggesting synergistic effects leading to increased pathogenicity, while others report competitive interactions between strains. Co-circulating AIV in a host can be influenced by many factors such as the specific viral strains involved, timing of infections, host species, and individual immune status. Additionally, co-infections complicate diagnosis and potentially impact the effectiveness of vaccination strategies in poultry. While significant progress has been made in understanding these dynamics, many aspects of avian influenza co-infections, including their frequency in natural settings and long-term ecological consequences, remain areas of active investigation. As our findings suggest, co-infections may be increasing over time as multiple AIV subtypes continue to circulate globally, which highlights the growing need for integrated PLM and phylogenetic approaches to track emerging co-infections and reassortment events across host species.

## Data Availability

The datasets presented in this study can be found in online repositories. The names of the repository/repositories and accession number(s) can be found here. GISAID Identifier: EPI_SET_250518gs DOI: https://doi.org/10.55876/gis8.250518gs. All genome sequences and associated metadata in this dataset are published in GISAID’s EpiFlu database. To view the contributors of each individual sequence with details such as accession number, Virus name, Collection date, Originating Lab and Submitting Lab and the list of Authors, visit 10.55876/gis8.250107tk.
