# Supplementary material for: Genomic-based biosurveillance for avian influenza: whole genome sequencing from wild mallards sampled during autumn migration in 2022–2023 reveals a high co-infection rate on migration stopover site in Georgia
Source: Front Microbiol. 2026 Jan 28;17:1735728. doi: 10.3389/fmicb.2026.1735728 (PMC12891182; doi:10.3389/fmicb.2026.1735728)
Supplement: Supplementary file 2 [file Table_2.pdf]

Supplementary Table 2. GenBank accession numbers of reference sequences used for read mapping and segment-specific analyses of Georgian avian influenza virus genomes.

Segment	GenBank accession numbers
HA	MK237717; MK237264; MK236653; MK237830; MK236649; MK995694; MK237762; MK995833; MH068663; MK237255; MK236848; MK995801; MN210139; MK928228; KY635719; MN210187
NA	MN210286; MK236743; MN210149; MK237631; MK830105; MK995835; MK237963; MK236673; MK995696
